# Development strategy and lessons learned for a 10-valent pneumococcal conjugate vaccine (*PNEUMOSIL®*)

**DOI:** 10.1080/21645515.2021.1874219

**Published:** 2021-02-24

**Authors:** Mark R. Alderson, Vistasp Sethna, Lauren C. Newhouse, Steve Lamola, Rajeev Dhere

**Affiliations:** a Center for Vaccine Innovation and Access, PATH, Seattle, Washington, USA; b R&D Department, Serum Institute of India, Pvt. Ltd, Pune, India; c Medical Department, Serum Institute of India, Pvt. Ltd., Pune, India

**Keywords:** Pneumococcus, pneumococcal conjugate vaccine, vaccine development, low- and middle-income countries, collaboration, SIIPL, PATH

## Abstract

Pneumococcal conjugate vaccines (PCVs) have proven to be the best way to prevent severe childhood pneumococcal disease but until recently have been difficult for many countries to afford sustainably. In 2008, the Serum Institute of India, Pvt. Ltd. and PATH entered into a collaboration, funded in part by the Bill & Melinda Gates Foundation, to respond to this problem by developing a PCV designed to be affordable, accessible, and protective against the pneumococcal serotypes causing the most morbidity and mortality in low- and middle-income countries. The resulting 10-valent PCV (*PNEUMOSIL®*) received World Health Organization prequalification in December 2019 – making it just the third PCV to be certified as an option for Gavi, the Vaccine Alliance-eligible countries – and is being made available at a Gavi price of US$2/dose. The task of developing a state-of-the-art, yet lower-priced, PCV required public-private collaboration across geographies and yielded a variety of successes and learnings useful to the vaccine development field. Key among the learnings were factors related to manufacturing strategy and optimization, serotype selection, flexibility, early risk detection and mitigation, partner trust and continuity across similar-class products, complementary business philosophies, and early clarity of purpose.

## Introduction

Pneumonia remains the top infectious disease killer of children under five worldwide, with most mortality in low- and middle-income countries (LMICs).^1^ Pneumococcal conjugate vaccines (PCVs) have helped reduce severe childhood pneumonia and other pneumococcal diseases like meningitis, sepsis, and otitis media since first licensure in 2000.^2,3^ Relatively high PCV prices have, however, made access cost-prohibitive for many countries and only possible for low-income economies eligible for Gavi, the Vaccine Alliance financial support.^4^ Additionally, PCV financing disproportionately appropriates donor funds compared to other vaccines – approximately 43% of Gavi’s 2016–2020 vaccine expenditures and over 24% anticipated through 2025.^5,6^

**Figure 1.**
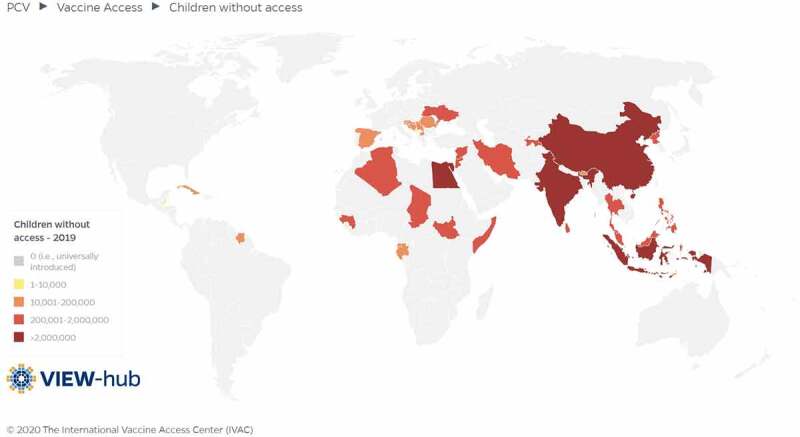
World map overview of the number ranges and locations of children without access to pneumococcal conjugate vaccines in 2019

PCVs are extremely complex to manufacture and, until 2019, only two multinational corporations had achieved World Health Organization (WHO) prequalification. This duopoly has limited supply and stifled competitive market forces that drive prices down. To assuage these dynamics, Gavi created the pneumococcal Advance Market Commitment (AMC) – a financing mechanism designed to incentivize PCV manufacturers to scale up supply and encourage additional manufacturers to develop PCVs suitable for Gavi-supported countries. Still, price and supply barriers leave over 55 million children without access to PCVs (Figure 1)^7^ – underscoring an urgency for more affordable vaccines that protect on par with existing ones, alleviate financial burdens on countries and donors, and foster sustainable, universal PCV access.

In 2008, Serum Institute of India, Pvt. Ltd. (SIIPL) and PATH initiated the development of such a PCV, with funding partly provided by the Bill & Melinda Gates Foundation. A decade later, the resulting 10-valent PCV – *PNEUMOSIL®* – is WHO-prequalified, AMC-approved, and licensed in India.^8^ *PNEUMOSIL’s* LMIC price is an unprecedented US$2 per dose – a reduction of over 30% from the Gavi-supported price for other PCVs and dramatically less than non-Gavi prices.^9^ It is also the first PCV designed specifically to target serotypes most likely to cause invasive pneumococcal disease (IPD) in Africa and Asia (the highest-burden regions), and Latin America and the Caribbean (LAC).^10^

Herein, we discuss factors contributing to *PNEUMOSIL’s* successful development, learnings, and future considerations. The purpose is to inform others seeking to accelerate the development, lower the costs, and preserve the quality of vaccines for high-burden, resource-limited populations.

### Collaboration structure and strategy

In 2006, PATH received a grant from the Bill & Melinda Gates Foundation for its pneumococcal vaccine project (PVP) to accelerate the development of affordable and accessible pneumococcal vaccines for LMICs. Initially, PVP’s target was protein vaccines but delays in development led to higher demand for a lower-cost PCV – shifting PVP’s top priority to affordable PCVs.

The 2008 SIIPL-PATH collaboration built upon an existing collaboration evaluating novel technologies for conjugate vaccines. The partners decided to focus on validated technologies to minimize risk and timelines to licensure. They also applied learnings from a previous partnership with WHO and African countries that developed and introduced *MenAfriVac*® – a low-priced *Neisseria meningitidis* serogroup A conjugate vaccine that eliminated meningitis A epidemics in Africa’s meningitis belt.^11^

*PNEUMOSIL’s* developmentgoal was two-fold: WHO prequalification to catalyze improved LMIC PCV access and Indian marketing authorization (MA). In a two-staged global access strategy, initial collaboration agreements adhered to broad provisions that ensured appropriate pricing and distribution, with terms specifying general affordability and supply. Later, long-term, large-scale clinical development resourcing (from SIIPL in India and the Bill & Melinda Gates Foundation in Africa) afforded sufficient confidence in program continuity for more concrete global access terms to be established using tiered pricing, including a target price of 2 USD per dose for LMICs, contingent upon demand.

#### Serotype selection

The challenge was to develop a more affordable PCV with comparable performance, quality, and serotype coverage in LMICs as existing vaccines. Pneumococcal Global Serotype Project and other disease burden data helped prioritize serotypes causing the highest pediatric IPD incidence in Africa, Asia, and LAC. Ultimately, ten serotypes (1, 5, 6A, 6B, 7F, 9V, 14, 19A, 19F, 23F) were chosen for optimal balance of cost, coverage, and competitiveness with existing PCVs.^10^ Accordingly, *PNEUMOSIL’s* theoretical cumulative coverage (71%) in these regions is comparable to the other WHO-prequalified 10- and 13-valent PCVs (66% [*Synflorix™*] and 76% [*Prevnar 13®*], respectively).^10^ Serotypes 6A and 19A were emerging as important causes of IPD in target regions, so were critical inclusions to ensure that *PNEUMOSIL* matched or exceeded other PCVs for serotype coverage.

#### Formulation, presentation, and manufacturing optimization

Decisions around dose level, carrier protein, conjugation technology, adjuvant (at the lowest possible concentration to minimize reactogenicity), and preservative (for multi-dose formulation) were all based upon proven approaches to minimize risk. Additional strategic input from PVP’s consultants and scientific advisory board (SAB) was also considered. Vaccine specifications also followed WHO’s TRS 977 Annex 3 guidance and the AMC target product profile (TPP).

Importantly, SIIPL optimized three innovative manufacturing processes that substantially lowered costs while maintaining high vaccine quality – carrier protein production, polysaccharide production, and conjugation efficiency. A key innovation was early implementation and optimization of an efficient manufacturing process for the CRM_197_ carrier protein using Pfenex, Inc.’s recombinant expression technology. Another innovation involved optimizing an efficient conjugation process using CDAP (1-cyano-4-dimethylaminopyridinium tetrafluoroborate) chemistry in collaboration with Fina Biosolutions.

#### Preclinical development

*PNEUMOSIL* underwent seven toxicology studies in India in rats and rabbits with regulatory oversight by the Review Committee on Genetic Manipulation (RCGM) of India’s Department of Biotechnology. Single- and repeat-doses (even at >20 times the human dose) were well tolerated – findings similar to those with a licensed comparator PCV. Serotype-specific immune response assessment used an enzyme-linked immunosorbent assay (ELISA) to quantitate serotype-specific immunoglobulin G (IgG) and a multiplexed opsonophagocytic activity (MOPA) assay to estimate functional antibodies. Total IgG and functional antibody responses were equivalent to those of the licensed PCV comparator across all vaccine serotypes. Similar analyses with multiple lots after 1 year of manufacture showed comparable IgG and OPA antibody titers to those at the time of manufacture. Based on these results, RCGM approved the Indian national regulatory authority, the Drug Controller General of India (DCGI), to advance *PNEUMOSIL* into human studies.

#### An evolving clinical strategy

*PNEUMOSIL’s* clinical development plan evolved as circumstances changed (Table 1). Originally, SIIPL and PATH planned to partner on the trials required for Indian MA, which would constitute the basis for WHO prequalification. When it became clear that the Phase 1 trial in India was restricted to a safety study in adults from a regulatory perspective, the team adjusted strategy to conduct a parallel Phase 1/2 clinical trial in Africa to generate early safety and immunogenicity data in adults, toddlers, and infants (the target population). The team determined that the quickest way to WHO prequalification and Indian MA was to undertake both India and Africa clinical development in parallel. Doing so would de-risk the program from potential region-specific delays and generate clinical data from diverse populations. In the resulting separate but complementary programs, SIIPL led and funded the Indian studies for MA, and PATH (with a grant from the Bill & Melinda Gates Foundation) sponsored the African studies required for WHO prequalification. To maintain cohesion, the SIIPL and PATH teams drove the evolving clinical strategy across regulatory landscapes and technical consensus-building between the Indian national regulatory authority, key health agencies, and WHO. The strategy shift meant that WHO prequalification would now be based on a Notice of Compliance (NOC) for export from DCGI – an approach informed by consultations with Indian regulators and WHO.

**Table 1.** List of completed, ongoing, and planned *PNEUMOSIL®* clinical studies as of November 2020

Phase	Purpose	Population	Comparator	Sponsor	Location	Years	Status
1	Safety	Adults	Polysaccharide pneumococcal vaccine *(Pneumovax 23®)*	Serum Institute of India, Pvt. Ltd. (SIIPL)	India	2014–2015	Complete (results presented at ISPPD)
1/2	Safety; tolerability; immunogenicity (3 + 0 schedule)	Adults; toddlers; infants	*Pneumovax 23*; 13-valent PCV (*Prevnar 13®);* routine EPI vaccines	PATH	The Gambia	2015–2016	Complete (results published^12^)
2	Safety; tolerability; immunogenicity (catch-up schedule, two doses, 8 weeks apart)	Toddlers	*Prevnar 13*	SIIPL	India	2016–2017	Complete (results presented at ISPPD)
3	Safety; tolerability; immunogenicity; non-inferiority to licensed PCV; lot-to-lot consistency; noninterference with EPI vaccines (3 + 0 schedule; booster)	Infants	10-valent PCV (*Synflorix™);* routine EPI vaccines	PATH	The Gambia	2017–2018	Complete (results in press^13^)
3	Immunogenicity; safety; tolerability (3 + 0 schedule)	Infants	*Prevnar 13*; *Synflorix*	SIIPL	India	2019–2020	Complete (manuscript in preparation)
3	Safety; immunogenicity (2 + 1 schedule)	Infants	*Prevnar 13; Synflorix*; routine EPI vaccines	PATH	The Gambia	2019-	Ongoing
3	Immunogenicity; safety; tolerability (2 + 1 schedule)	Infants	*Prevnar 13*; *Synflorix*	SIIPL	India	2019	Ongoing

Definitions: PCV – pneumococcal conjugate vaccine; EPI – Expanded Program on Immunization; ISPPD = International Symposium on Pneumococci and Pneumococcal Diseases

The revised strategy’s advantages included preserving the pathway to Indian MA and introduction, minimizing time to WHO prequalification, achieving AMC eligibility and access for Gavi-eligible countries sooner,^14^ and generating robust late-stage clinical data in important representative target geographies.

#### Indian clinical studies (2014–2020)

SIIPL conducted a series of clinical studies to support Indian MA and Universal Immunization Program applications. Sites across India were selected primarily on SIIPL’s longstanding experience, vaccine trial expertise, and prior experience conducting large studies of PCVs with Indian licensure. The studies included Phase 1 (PCV-10-001) supported by contract research organization (CRO) SIRO Clinpharm Pvt. Ltd., Phase 2 (PCV-10-002), and Phase 3 (PCV-10-003). The CRO for the latter two studies was DiagnoSearch Life Sciences Pvt. Ltd. The WHO Reference Laboratory for Pneumococcal Serology at University College London (UCL) conducted serology testing (serotype-specific IgG ELISAs and MOPAs), with safety labs at Metropolis Healthcare Limited and DiagnoSearch Central Laboratory.

The Phase 1, first-in-human, single-dose study demonstrated acceptable safety and tolerability in 34 healthy adults 18–40 years old – an important trigger for initiating the African clinical program and advancing the Indian program into Phase 2. The Phase 2 in 114 healthy PCV-naive toddlers 12–15 months old used a 2-dose catch-up regimen and *Prevnar 13* as comparator. *PNEUMOSIL* demonstrated acceptable safety and tolerability and was immunogenic for all 10 vaccine serotypes – supporting entering Phase 3.

The multi-site Phase 3 trial enrolled 448 infants and used a 3 + 0 schedule (aligning with India’s Central Drugs Standard Control Organization guidelines and one of two WHO-approved schedules). It met all primary and secondary objectives (Table 2), with *PNEUMOSIL* demonstrating acceptable safety and tolerability and eliciting immune responses comparable to *Prevnar 13* and *Synflorix* for all 10 serotypes.

**Table 2.** Primary and secondary objectives and endpoints for Phase 3 clinical study in India to evaluate *PNEUMOSIL®* in a three-dose primary series

Objective	Objective description	Endpoints
Primary (immunogenicity)	To evaluate in a 3 + 0 schedule the serum IgG antibody responses [seroresponse rates and GMCs] to the 10 matched serotypes in *PNEUMOSIL* (1, 5, 6A, 6B, 7F, 9V, 14, 19A, 19F, 23F), alone and in comparison to antibody responses to these serotypes induced by *Prevnar 13.*	IgG responses 4 weeks after a 3-dose series, alone and in comparison to *Prevnar 13*: Percentage of subjects with serotype-specific IgG concentrations ≥ 0.35 μg/mL Serotype-specific IgG GMCs
Primary (safety/tolerability)	To assess the safety and tolerability of a 3 + 0 schedule of PNEUMOSIL, alone and in comparison to *Prevnar 13*.	Alone and in comparison to *Prevnar 13*: Number and severity of solicited local and systemic AEs during the first 7 days post all 3 doses of the study vaccine Number, severity, and relatedness of all unsolicited AEs through 4 weeks post all 3 doses of the study vaccine Number, severity, and relatedness of SAEs with onset through 4 weeks after a 3-dose series
Secondary (immunogenicity)	To evaluate in a 3 + 0 schedule the serum functional antibody responses [seroresponse rates and GMTs] to the 10 matched serotypes in *PNEUMOSIL* as measured by OPA, alone, and in comparison to the functional antibody responses to these serotypes induced by *Prevnar 13* (subset of subjects).	4 weeks after a 3-dose series: OPA responses, alone and in comparison to *Prevnar 13* Percentage of subjects with serotype-specific serum OPA titers ≥ 1:8 Serotype-specific serum OPA GMTs
	To evaluate in a 3 + 0 schedule the serum IgG antibody responses (seroresponse rates and GMCs) of the 10 serotypes in *PNEUMOSIL* (1, 5, 6A, 6B, 7F, 9V, 14, 19A, 19F, 23F), alone and in comparison to *Synflorix™* for the 8 matched serotypes (1, 5, 6B, 7F, 9V, 14, 19F, 23F) and for 6A and 19A the lowest responder in *Synflorix.*	4 weeks after a 3-dose series: IgG responses, alone and in comparison to *Synflorix* Percentage of subjects with serotype-specific IgG concentrations ≥ 0.35 μg/mL Serotype-specific IgG GMCs
	To evaluate in a 3 + 0 schedule the serum functional antibody responses (seroresponse rates and GMTs) of the 10 serotypes in *PNEUMOSIL* (1, 5, 6A, 6B, 7F, 9V, 14, 19A, 19F, 23F), alone and in comparison to *Synflorix* for the 8 matched serotypes (1, 5, 6B, 7F, 9V, 14, 19F, 23F) and for 6A and 19A the lowest responder in *Synflorix* (subset of subjects).	4 weeks after a 3-dose series: OPA responses, alone and in comparison to *Synflorix* Percentage of subjects with serotype-specific serum OPA titers ≥ 1:8 Serotype-specific serum OPA GMTs

Definitions: IgG – immunoglobulin G; GMC – geometric mean concentration; Hib – *Haemophilus influenzae* type B; AE – adverse event; SAE – serious adverse event; OPA – opsonophagocytic assay; GMT – geometric mean titer

##### African clinical studies (2015–2020)

After considering various locations, a site run by MRC Unit in The Gambia – part of the London School of Hygiene and Tropical Medicine (LSHTM) – was selected. Factors favoring The Gambia included previous PATH/SIIPL experience with the MRC site, the country’s expertise in pneumococcal disease and vaccines, and large efficacy study experience with a PCV.^15^

Studies included a Phase 1/2 age de-escalation study and a non-inferiority Phase 3 trial required for WHO prequalification. CRO FHI Clinical supported both trials. Pneumococcal serology occurred at UCL while other concomitant vaccine laboratory testing occurred at Public Health England, Q^2^ Solutions, Cincinnati Children’s Hospital and Medical Center, and the US Centers for Disease Control and Prevention.

The Phase 1/2 trial evaluated *PNEUMOSIL*, first, in 34 healthy PCV-naïve adults 18–40 years old, then in 112 PCV-primed children 12–15 months old, and finally in 200 PCV-naïve infants 6–8 weeks old using a 3 + 1 schedule. The comparator for infants and children was *Prevnar 13. PNEUMOSIL* demonstrated an acceptable safety and tolerability profile and was immunogenic for all 10 vaccine serotypes – supporting entering Phase 3.^12^

The Gambian, multi-site Phase 3 enrolled 2,250 infants to receive either *PNEUMOSIL* or *Synflorix* in a primary 3 + 0 schedule, with a subset of participants receiving a booster dose. *PNEUMOSIL* met all primary and secondary endpoints, including safety and tolerability, immunological non-inferiority to *Synflorix* per WHO specifications (TRS 977 Annex 3) for all 10 vaccine serotypes, lot-to-lot consistency, and noninterference with routine childhood immunizations (Table 3).^13^ Immune response persistence was demonstrated at 10 months of age following the 3-dose priming series in the Gambian Phase 1/2 study^12^ and 12 months following the booster immunization in the Gambian Phase 3 study.^13^

**Table 3.** Primary and secondary objectives and endpoints for Phase 3 clinical study in The Gambia to evaluate *PNEUMOSIL®* in a 3-dose primary series with booster (3 + 1)^13^

Objective	Objective description	Endpoints
Primary (immunogenicity)	Demonstrate equivalent immune responses to the 10 vaccine serotypes across 3 different lots when measured 4 weeks post dose 3.	*Lot consistency* Serotype-specific IgG GMC measured 4 weeks post dose 3
	Demonstrate non-inferior immune responses for at least 7 of the 10 vaccine serotypes in comparison to matched serotypes or lowest responder in *Synflorix™.*	*Non-inferiority* % subjects with serotype-specific IgG concentrations ≥ 0.35 µg/mL measured 4 weeks post dose 3 Serotype-specific IgG GMCs measured 4 weeks post dose 3
	Demonstrate that immune responses induced by routine pediatric vaccines (pentavalent and polio) when co-administered with *PNEUMOSIL* are non-inferior to those induced when co-administered with *Synflorix.*	*Noninterference* % subjects with anti-diphtheria and anti-tetanus toxoid IgG concentrations ≥0.1 IU/mL measured 4 weeks post-dose 3% subjects with anti-Hepatitis B surface antigen IgG concentration ≥ 10 mIU/mL measured 4 weeks post-dose 3% subjects with anti-Hib IgG concentration ≥0.15 µg/mL measured 4 weeks post dose 3 Anti-pertussis IgG GMCs measured 4 weeks post-dose 3% subjects with anti-poliovirus 1 and 3 neutralizing antibody titers ≥1:8 measured 4 weeks post dose 3
Primary (safety/tolerability)	Demonstrate acceptable safety and tolerability profile for PNEUMOSIL administered as a 3-dose primary series and booster dose, and when co-administered with routine EPI vaccines.	# and severity of solicited local and systemic AEs during first 7 days after vaccination #, severity, and relatedness of all AEs and SAEs through the entire study period
Secondary (immunogenicity)	Demonstrate that immune responses to serotypes 6A and 19A in *PNEUMOSIL* are superior to the cross-reactive responses to these serotypes induced by *Synflorix.*	*Superiority* % subjects with serotype-specific IgG concentrations ≥ 0.35 µg/mL measured 4 weeks post dose 3 Serotype-specific IgG GMCs measured 4 weeks post dose 3
	Evaluate functional serotype-specific antibody responses induced by *PNEUMOSIL* in comparison to *Synflorix.*	*Functional response* % subjects with OPA titer ≥1:8 measured 4 weeks post-dose 3 OPA GMT measured 4 weeks post-dose 3
	Evaluate the booster response (antibody concentrations and functional responses) to *PNEUMOSIL* in comparison to *Synflorix.*	*Boostability* Ratio of IgG GMCs measured 4 weeks post dose 4 to IgG GMCs measured 4 weeks post dose 3 Ratio of OPA GMTs measured 4 weeks post dose 4 to OPA GMTs measured 4 weeks post dose 3
	Demonstrate that immune response induced by measles-rubella and yellow fever vaccines when co-administered with a booster dose of *PNEUMOSIL* are non-inferior to those induced by these vaccines when co-administered with a *Synflorix* booster dose.	*Noninterference* % subjects with anti-measles IgG concentration ≥ 150 mIU/mL measured 4 weeks post dose 3 % subjects with anti-yellow fever neutralizing antibody titers ≥ 1:8 measured 4 weeks post dose 4 % subjects with anti-rubella IgG concentration ≥4 IU/mL

Definitions: IgG – immunoglobulin G; GMC – geometric mean concentration; Hib – *Haemophilus influenzae* type B; AE – adverse event; SAE – serious adverse event; OPA – opsonophagocytic activity; GMT – geometric mean titer

The African and Indian clinical programs achieved a complementary data package that provided a comprehensive picture of *PNEUMOSIL’s* performance relative to both WHO-prequalified PCVs. Using *Synflorix* as comparator in the Gambian Phase 3 added value because *PNEUMOSIL* had been evaluated in India and The Gambia with *Prevnar 13* as comparator. Furthermore, *Synflorix* was highly efficacious against vaccine-type IPD, pneumonia, and otitis media in infants and children in randomized controlled trials^16,17^ and effective against IPD and pneumonia in infants in sub-Saharan Africa.^18,19^ (Regarding *Prevnar 13* and *Synflorix*, a recent WHO position paper stated that “the currently available evidence does not point to a difference between the two vaccines in their net impact on the overall pneumococcal disease burden.”)^20^ Strengthening comprehensiveness, the Indian Phase 3 evaluated *PNEUMOSIL* head-to-head against *Prevnar 13* and *Synflorix* (manuscript in preparation).

#### Approval milestones and looking forward

*PNEUMOSIL* received the Indian Export NOC in December 2018, after which SIIPL applied for WHO prequalification in January 2019 with support from PATH, FHI Clinical, and 4Clinics medical writers. *PNEUMOSIL* received WHO prequalification in December 2019, an AMC supply agreement in June 2020, and Indian MA in July 2020.^21,22^

As a result, *PNEUMOSIL* is now an option for LMICs to consider for PCV introduction or program switch. Efforts are underway to share information about this new vaccine with LMICs and to continue evaluating the vaccine to inform public health decisions. SIIPL will conduct a post-licensure nasopharyngeal carriage study to obtain data on vaccine effectiveness required by WHO as a condition of prequalification. Ongoing clinical studies are evaluating *PNEUMOSIL* in the other WHO-recommended schedule (2 + 1) – one by SIIPL in India to meet MA regulatory requirements and inform uptake and the other by PATH and MRC Unit The Gambia at LSHTM to augment data for LMIC decision-making. A 2 + 1 schedule could be beneficial because the third vaccination at 9 to 18 months of age provides a better boost to protective antibodies and potentially contributes to stronger herd protection.^20,23^

### Keys to success and lessons learned

To inform others seeking to accelerate vaccine development for LMICs, *PNEUMOSIL* development yielded the following keys to success and learnings:

*Manufacturing optimization to reduce cost of goods sold (COGS) and serotype selection are key for reducing PCV price*. Since PCVs are among the most complex and expensive vaccines to manufacture, optimizing manufacturing processes is difficult and the business risks of changing processes can be disincentivizing. Developed from scratch, however, *PNEUMOSIL* was not beholden to these dynamics. Manufacturing optimization could proceed in a manner that improved the vaccine’s quality/performance, reduced its COGS, and supported its high-volume/low-price value proposition. The decision to include only serotypes most relevant for LMICs (especially 6A and 19A) while omitting those less applicable proved invaluable to *PNEUMOSIL’s* advantageous balance of coverage, performance, and cost for LMICs. Since serotypes vary by region and each adds expense, risk, and time to development and manufacturing, *PNEUMOSIL’s* strategy achieved the best of both worlds – coverage and immunity comparable to prequalified PCVs (if not superior in some cases) at a lower price point.

*Regulatory management and clinical consensus-building helped detect and mitigate risks early*. Taking a vaccine candidate into humans comes with risk; therefore, having safeguards and the necessary expertise to agilely resolve issues is important. A key to *PNEUMOSIL’s* success was adjusting the clinical and regulatory strategy when roadblocks arose, which was possible because the skillsets were in place to recognize risks and mitigate them. SIIPL and PATH’s cross-functional team of experts identified the regulatory landscape’s evolution in India early (e.g., through SIIPL’s engagement with DCGI and ministry agencies and multi-agency consensus meetings), which otherwise could have incurred debilitating delays for the program if undetected. The *PNEUMOSIL* program was significantly ahead of the PCV-specific regulatory and industry curve at the time and, therefore, needed intense negotiations to build consensus around the trans-continental program. The team worked together to consider scenarios and build consensus around regulatory options internally and, then, coordinated with regulators in India and WHO to identify an optimal alternative and lay a foundation to develop an appropriate clinical strategy.

*Clinical team flexibility enabled a smooth transition to a dual pathway*. The cross-geographical clinical teams’ flexibility to react to and execute a new strategy enabled the parallel India–Gambia clinical plan to come to fruition. The willingness to change tack and adjust to fit circumstances saved valuable time and enabled *PNEUMOSIL’s* market entry earlier than otherwise possible. Challenges, however, included added layers of complexity involved keeping two simultaneous geographical efforts coordinated and tracking requirements for DCGI, The Gambia’s Ethics Committee, data safety monitoring boards, WHO prequalification, and other elements. Frequent check-ins (with regular multi-polar regulatory engagements) helped mitigate these challenges.

*Value of partner continuity and trust*. Partnering across multiple projects and similar-class products built upon expertise and enabled efficiencies. The fact that several of *PNEUMOSIL’s* collaborators had previously worked together on vaccine development enabled learnings to be applied to *PNEUMOSIL* and leveraged trusting relationships. Expertise from groups like UCL, PVP’s SAB, the UK National Institute of Biological Standards and Control, and consultants further strengthened the ability to develop and evaluate the vaccine successfully. Strength of partnership also helped expedite the WHO prequalification and Indian licensure process. Additionally, early large-scale and long-term financial and resource commitments from SIIPL and the Bill & Melinda Gates Foundation provided leeway needed for end-to-end line of sight, therefore reducing risk and streamlining effort. The team also aligned with advocacy groups to share evidence, raise awareness, and strengthen the enabling environment for new PCVs. Overall, partner trust and continuity-conserved resources, accelerated timelines, tapped established lines of communication, reduced risk, and streamlined administrative process. (Table 4.)

**Table 4.** List of collaborators contributing to the development of *PNEUMOSIL®.*

Collaborator	Role
PATH	Coordinator for PATH pneumococcal vaccine project; subject matter expertise; design and sponsor for Africa clinical trials; regulatory facilitation for World Health Organization prequalification
Serum Institute of India, Pvt. Ltd	*PNEUMOSIL* developer and manufacturer; India clinical trial design, sponsor, and conduct; regulatory facilitation for Indian export license and marketing authorization
Bill & Melinda Gates Foundation	Funder and subject matter expertise
David Goldblatt and the WHO Reference Laboratory for Pneumococcal Serology at the Great Ormond Street Institute of Child Health, University College London	Clinical serology (serotype-specific immunoglobulin G enzyme-linked immunosorbent assays and opsonophagocytic killing assays) and subject matter expertise
MRC Unit The Gambia at the London School of Hygiene and Tropical Medicine	Clinical trial site and implementor (Africa)
KEM Hospital and Research Center, Vadu; Hamdard Institute of Medical Sciences and Research and Associated Hakeem Abdul Hameed Centenary Hospital, New Delhi; Sri Ramachandra Hospital, Chennai; Bharati Vidyapeeth, Pune Institute of Child Health, Kolkata; Christian Medical College, Vellore; Shri Maharaja Gulab Sing Hospital, Government Medical College, Jammu; KLES Dr. Prabhakar Kore Hospital & MRC, Belgavi, KEM Hospital, Mumbai	Clinical trial sites and implementors (India)
FHI Clinical	Contract research organization (Africa)
DiagnoSearch Life Sciences Pvt. Ltd.	Contract research organization (India)
SIRO Clinpharm Pvt. Ltd.	Contract research organization (India)
Public Health England	Concomitant vaccine laboratory testing
Cincinnati Children’s Hospital Medical Center	Concomitant vaccine laboratory testing
Q^2^ Solutions	Concomitant vaccine laboratory testing
UK National Institute of Biological Standards and Control	Vaccine and manufacturing intermediate quality control assays
US Centers for Disease Control and Prevention	Concomitant vaccine laboratory testing
FinaBiosolutions LLC	Conjugation technology transfer and support
Pfenex, Inc.	CRM_197_ manufacturing technology
4Clinics	Medical writing
PATH pneumococcal vaccine project (PVP) scientific advisory board	Technical advisory consultation and subject matter expertise
John Hennessey	Consultant
Neil Ravenscroft	Consultant
Craig Laferriere	Consultant

*Complementary philosophies and early clarity of purpose kept project on target*. Commonalities between PATH’s PVP goal and SIIPL’s philosophy of producing high-quality vaccines at high volume and low prices for LMIC access were critical to achieving early clarity of purpose for *PNEUMOSIL*. Both organizations agreed at the outset that the PCV should be affordable for children most in need. Likewise, agreeing on the value proposition, TPP, and global access principles were essential starting points that reduced downstream risk and ensured a fit-for-purpose product.

## Conclusion

*PNEUMOSIL’s* market entry is an important milestone toward enhancing the world’s arsenal of state-of-the-art PCVs and alleviating the price and supply barriers that have historically hindered or precluded sustainable access for LMICs. It ends a decade-long drought in new PCV suppliers and could reshape the marketplace by providing a more affordable option of comparable quality and potential impact. For Gavi and the countries it supports, *PNEUMOSIL’s* 30% savings over other PCVs means public health funds can go farther against pneumococcal disease and other health priorities. This more cost-efficient option is also important for non-Gavi-supported LMICs, who face paying for PCVs themselves, often at elevated prices (e.g., 12.83–14.50 USD per dose in non-Gavi Pan American Health Organization countries).^24^

*PNEUMOSIL* constitutes not only a technical *tour de force* for SIIPL as only the third vaccine manufacturer to WHO-prequalify a PCV, it marks a turning point that could lead to dramatic public health impact and help put PCVs within reach for children still without affordable access. The journey would not have been possible without a diversity of like-minded dedicated researchers, manufacturers, governments, multilaterals, regulators, laboratories, non-governmental organizations, and foundations across continents that provided input, debate, and manpower on decisions along the way8
